# The Effect of Parenting Attitude on the Life Satisfaction of Early Adolescents and Their Parents: A Multi-Group Path Analysis through Ego-Resilience

**Published:** 2019-03

**Authors:** Haeyoung LEE, Eunmi LEE

**Affiliations:** 1. College of Nursing, Chung-Ang University, Seoul, Korea; 2. Department of Nursing, Research Institute for Basic Sciences, Hoseo University, Asan, Korea

**Keywords:** Parenting attitude, Life satisfaction, Ego-resilience, Early adolescents

## Abstract

**Background:**

This study aimed to identify the effects of parenting attitude on life satisfaction and to analyze difference according to household income level.

**Methods:**

Data from 1977 adolescents participating in Korean Children and Adolescents Panel Survey V (2013) was analyzed. As a method of analysis, multi-group path analysis was performed.

**Results:**

Positive parenting attitude had a significant influence on both resilience and life satisfaction for adolescents, while it only influenced life satisfaction for parents. The effects of parenting attitude on life satisfaction of early adolescents were not different according to income level; life satisfaction of parents was different according to income level. Further, positive parenting attitude had a significant effect on life satisfaction of parents when they have lower income than average.

**Conclusion:**

Positive parenting has an effect on the psychological security of early adolescents, and the higher adolescents perceive their parents’ supports, the higher their life satisfaction is. Therefore, parenting attitude should be considered fully in the development of nursing interventions, in which physical and psychological approach to adolescents is important.

## Introduction

Life satisfaction includes one’s cognitive appraisal of their satisfaction levels in various areas of one’s own life (1) and indicates that one has lived life well, and is satisfied. It is affected by subjectively perceived discrepancies in income, rather than actual differences (2). Furthermore, life satisfaction is high in countries where household income is high, and the income gap index is low (3). It is necessary, however, to examine income differences further since life satisfaction is influenced by both objective and subjective income (2,3).

Parenting attitude refers to the attitudes and behaviors parents express towards raising their adolescents, which influence not only adolescents’ personality and behavior, but also cognitive and emotional development (4,5). When parents’ parenting attitude is more effective and receptive, adolescents are active, extroverted, independent, and their social adaptability is high (6). When parents are rejecting, controlling, under-supervising, negligent or intrusive, and inconsistent, adolescents’ depression, aggression, and antisocial problem behaviors increase (7–9). Such parenting attitudes have been significantly related to family composition or socioeconomic status, and personal factors of parents or adolescents (10).

In general, parents are more likely to display a positive parenting attitude when their economic level is high and a negative parenting attitude when their economic level is low (11). Furthermore, parents’ financial difficulties can cause negative thoughts or aggressiveness and negatively influence adolescents’ social, emotional, and academic development due to the use of corporal punishment, coercion, and hostile parenting behaviors (12,13).

Life satisfaction is a cognitive and judgmental process (14). Life satisfaction is not the state of an invariant process, but changes according to an individual’s internal and environmental factors (15). Accordingly, the factors that affect life satisfaction can, in general, include not only personal factors such as emotional and psychological status, but also sociodemographic background, family, school, and community variables (16). Among those factors, positive parenting attitude has been found to increase parental role satisfaction and the higher the parenting satisfaction, the higher the life satisfaction (17). Furthermore, negative parenting attitude such as parental over-expectation, intrusiveness, and strict control have been reported to decrease adolescents’ life satisfaction (18).

Ego-resilience is the ability to adapt to maintain functioning and recover from negative emotional experiences by flexibly adjusting one’s level of self-control in a changing social environment and internal and external stress situations (19). Adolescents maintain their psychological stability well when ego-resilience is high while low egoresilience may cause adolescents to experience stress and anxiety (20). The more positive the parenting attitude, the higher the ego-resilience of adolescents (21). Parents’ overprotection and their own ego-resilience, however, have been reported to be unrelated to adolescents’ egoresilience (22).

The influence of parenting attitude on the life satisfaction of adolescents has been reported in many studies; however, the influence of parenting styles on the life satisfaction of parents is not well understood because only a few studies examined ego-resilience as a mediating variable (17–19). Accordingly, in the present study, we investigated the influence of parents’ positive parenting attitude (e.g., supervision, affection, and rational explanation) and negative parenting attitude (e.g., inconsistency, over-expectation, and intrusiveness) on the life satisfaction of parents and adolescents using a multi-group path analysis with adolescents’ ego-resilience acting as a mediator and differences in life satisfaction according to household income level.

## Materials and Methods

### Study population

Participants were selected from the data in the Korean Children and Youth Panel Survey (KCYPS). The KCYPS was conducted with 7071 adolescents in three panels sampled in 2010. Data from one of the three panels was used. Data was collected from both the 7th-grade students and their parents at the same time. There were 2,378 participants; however, only the data from 1,977 participants were analyzed after excluding missing data. In other words, data from 1,977 pair of participants were analyzed.

### Measures

#### Parenting attitude

For parenting attitude, items were selected from the scale by Heo, which includes supervision (3 items), affection (4 items), and rational explanation (3 items) that comprised of positive parenting attitude; inconsistency (3 items), over-expectation (4 items), and intrusiveness (4 items) were the components of negative parenting attitude (23). All items were answered using a 4-point Likert scale (1–4). Higher scores obtained by reverse scoring indicated elevated levels of positive and negative parenting attitudes.

#### Ego-resilience

The ego-resilience scale was developed, and modified by Yu and Sim (24, 25). The scale comprised 14 items, and each item was rated on a 4-point Likert scale (1–4). Higher scores obtained by reverse scoring indicated higher resilience.

#### Life satisfaction

Life satisfaction was measured by three items from the KCYPS, which include the level of joy felt about one’s own life (“I am happy to live”), the level of worry (“I do not have much to worry”), and thinking that life is happy (“I think I have a happy life”). The scale was answered using a 4-point Likert scale (1–4), and higher scores signify higher satisfaction with one’s own life. Both parent and adolescent’s life satisfaction were measured.

#### Statistical analyses

**Fig. 1:**
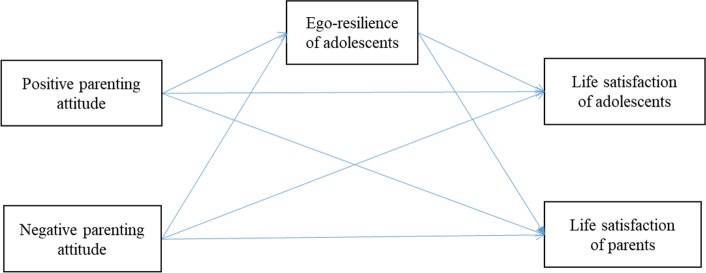
Hypothesized path model

Data were analyzed using PASW Statistics 23.0 and AMOS 20.0, and statistical tests were performed at the significance level of *P*<0.05. Cross-sectional weights were applied to the data since only one-year data were used. Differences among variables according to annual household income were tested using a two-sample t-test and χ^2^ test. Pearson’s correlation coefficients were used to analyze correlations among the variables. Goodness-of-fit of the hypothetical model (Fig. 1) was tested. Comparisons were made between the goodness-of-fit of the full model and the multi-group model by conducting multi-group path analysis according to annual household income.

Parenting attitude is closely related to the economic level. Therefore, the model has divided into two groups: 1) the average to above average income group, and 2) the below average income group, based on the average income in Korea (26). To verify the moderating effect of egoresilience, the direct effect, indirect effect, and total effect of each variable that influences the level of life satisfaction of both parents and adolescents were analyzed in the modified full model, the average and above average income model, and the below average income model.

The Institutional Review Board of the Hoseo University (1041231-161018-HR-048-01) approved this study.

## Results

The annual average household income was 4,664.62 South Korean won (KRW), and based on the average household income in 2013 (KRW 4,163.22), 981 participants (49.6%) had a below average income while 996 participants (50.4%) had an average to above average income.

### Differences in general characteristics and major variables according to income level

Differences in the general characteristics of the participants and major variables were significant according to income level and the relationship between guardians and adolescents, fathers’ and mothers’ highest education level and occupation, family composition, supervision in positive parenting attitude, affection, inconsistency in negative parenting attitude, ego-resilience, and parents’ life satisfaction (Table 1).

**Table 1:** Characteristics and differences in variables according to average income (N = 1,977)

***Variables***		***Below Average income***	***Above Average income***	***χ^2^***	***P***
		***n***	***n***		
Sex	Male	528	508	1.574	.224
	Female	453	488		
Guardian’s relationship with adolescent	Mother	818	885	20.022	<.01^**^
	Father	137	105		
	Grandmother	18	6		
	Grandfather	3	0		
	Brothers and sisters	1	0		
	Relative	4	0		
Father’s education level	Below middle school graduate	44	2	274.244	<.01^**^
	High school graduate	467	247		
	college graduate	106	92		
	University graduate	237	559		
	Graduate school	20	88		
Mother’s education level	Below middle school graduate	44	2	256.692	<.01^**^
	High school graduate	467	247		
	college graduate	106	92		
	University graduate	237	559		
	Graduate school	20	88		
Father’s job	Managers	58	153	137.604	<.01^**^
	Professionals and related workers	78	151		
	Clerks	103	203		
	Service workers	116	133		
	Sales workers	86	78		
	Skilled agricultural, forestry and fishery workers	24	16		
	Craft and related trades workers	139	92		
	Equipment, machine operating and assembling workers	139	95		
	Elementary workers	95	44		
	Armed forces	2	14		
Mother’s job	Managers	2	21	135.934	<.01^**^
	Professionals and related Workers	107	251		
	Clerks	68	136		
	Service workers	129	107		
	Sales workers	116	88		
	Skilled agricultural, forestry and fishery workers	13	5		
	Craft and related trades workers	26	9		
	Equipment, machine operating and assembling workers	17	19		
	Elementary workers	76	22		
Family composition	Parents + adolescents	788	932	121.095	<.01^**^
	Single parent + adolescents	106	11		
	Single grandparent + adolescents	9	0		
	Single grandparent + parents + adolescents	41	47		
	Single grandparent + single parent + adolescents	34	6		
	Others	3	0		
		***M ± SD***	***M ± SD***	***t***	***P***
Positive parenting attitude	Supervision	3.24 ± 0.593	3.37 ± 0.518	5.264	< .01^**^
	Affection	3.13 ± 0.582	3.21 ± 0.561	3.296	.01^**^
	Rational explanation	2.97 ± 0.604	3.00 ± 0.602	1.125	.261
Negative parenting attitude	Inconsistency	2.40 ± 0.689	2.28 ± 0.692	3.664	< .01^**^
	Over-expectation	2.62 ± 0.622	2.58 ± 0.634	1.550	.121
	Intrusiveness	2.34 ± 0.673	2.29 ± 0.678	1.397	.163
Ego-resilience		2.98 ± 0.474	3.03 ± 0.426	2.437	.015^*^
Life satisfaction of adolescent		3.14 ± 0.656	3.18 ± 0.606	1.352	.176
Life satisfaction of parent		2.84 ± 0.593	3.09 ± 0.465	10.567	< .01^**^

*Note: M*: mean, *SD*: standard deviation;

* *P*<0.05,

** *P*<0.01

### Correlation between main variables

Supervision, affection, and rational explanation in positive parenting attitude showed significant positive correlations with ego-resilience, adolescents’ life satisfaction in both groups. Supervision, affection, and rational explanation in positive parenting attitude were significantly correlated to life satisfaction of parents within the group earning less than average. Among negative parenting attitudes, excessive expectation was positively correlated to ego resiliency of children, and over-involvement was negatively correlated to it. In addition, ego resiliency of children had a significant positive correlation with adolescents’ life satisfaction. While the group with less-than-average income showed a significant positive correlation between parents’ life satisfaction and all aspects of positive parenting attitudes, the group with above-average income did not have any significant associations between life satisfaction and positive parenting attitudes (Table 2).

**Table 2:** Correlations for measured variables in the two groups

***Variable***		***Positive parenting attitude***			***Negative parenting attitude***			***Ego-resilience***	***Life satisfaction of adolescent***	***Life satisfaction of parent***
		***Supervision***	***Affection***	***Rational explanation***	***Inconsistency***	***Over-expectation***	***Intrusiveness***			
Positive parenting attitude	Supervision	1	.516^**^	.415^**^	−.181^**^	.023	−.141^**^	.320^**^	.342^**^	.015
	Affection	.550^**^	1	.673^**^	−.304^**^	−.078^*^	−.280^**^	.398^**^	.484^**^	.029
	Rational explanation	.450^**^	.692^**^	1	−.215^**^	−.049	−.156^**^	.345^**^	.371^**^	.006
Negative parenting attitude	Inconsistency	−.06	−.146^**^	−.069	1	.518^**^	.626^**^	−.027	−.141^**^	−.04
	Over-expectation	.182^**^	.063^**^	.085^**^	.561^**^	1	.701^**^	.148^**^	.045	.027
	Intrusiveness	−.021	−.147^**^	−.045	.628^**^	.686^**^	1	−.01	−.105^**^	.005
Ego-resilience		.316^**^	.390^**^	.317^**^	.023	.161^**^	.069^*^	1	.483^**^	.065
Life satisfaction of adolescent		.269^**^	.391^**^	.287^**^	−.044	.078^**^	−.063^*^	.473^**^	1	.033
Life satisfaction of parent		.122^**^	.142^**^	.109^**^	−.014	−.001	−.064	.075^**^	.123^**^	1

*Note:* Correlations above the diagonal are for the group above average income (n=996); Those below the diagonal are for the group below average income (n=981).

* *P*<0.05,

** *P*<0.01

### Multi-group path analysis

**Fig. 2:**
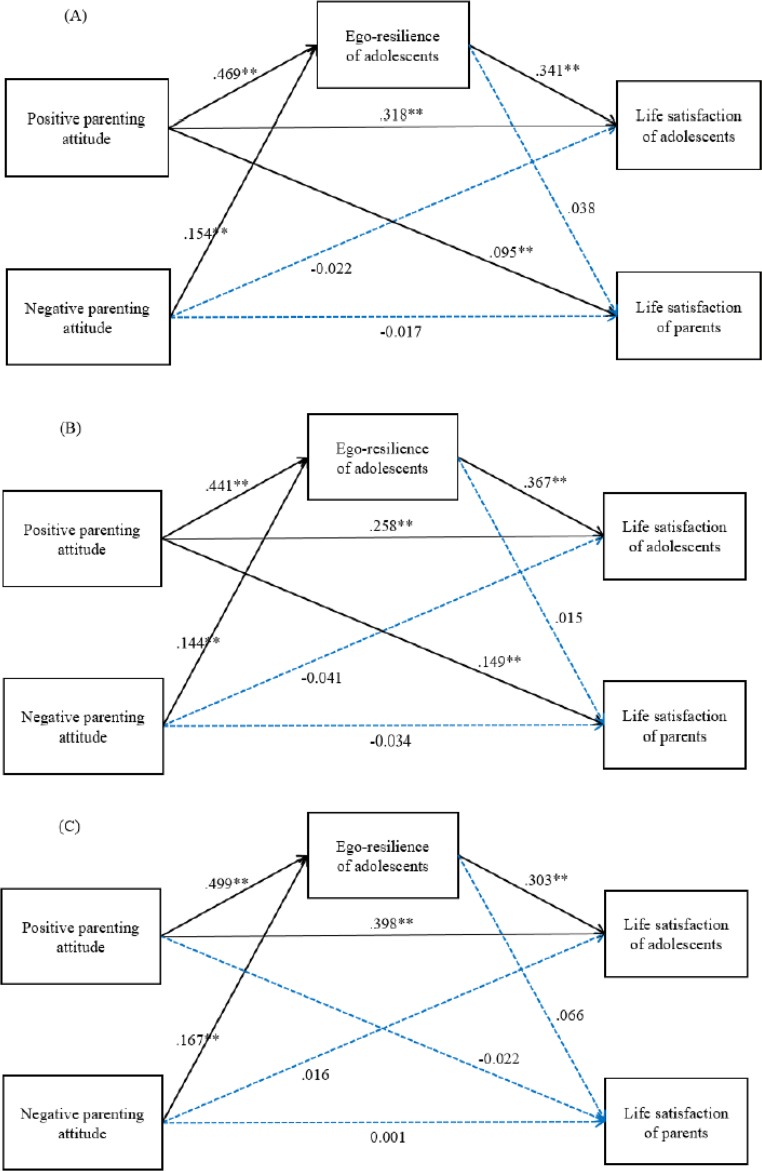
Final study model: total (A), below average income (B), above average income (C)

Standardized coefficients were used to compare the changes of path coefficients in the full model and multi-group path model. Because the results of goodness-of-fit test of the full path model, which was a hypothetical model, and the multi-group path model showed χ^2^/*df* values of two or greater and the root mean square error of approximation (RMSEA) value was greater than 0.08, the hypothetical model (Fig. 1) was modified by removing “over-expectation.” Since the modified model explained the hypothetical model better with a χ^2^/*df* value close to two, RMSEA value less than 0.08, and CFI value 0.991, it was chosen as the final model (Table 3, Fig. 2). When the Chi-square change (δ_χ^2^_) is statistically significant, the structural invariances of the two models are different (27). In the case of the revised hypothesis, it is necessary to conduct separate analysis for each group by making a difference between the groups earning average income or more and income below the average. Therefore, we analyzed how each model fits (Table 3), as a result, both groups showed adequate conformity. For the full model of ego-resilience (Fig. 2A), the direct effect (β = .469) and the total effect (β = .154) of positive parenting attitude (β = .469) and negative parenting attitude (β = .154) were significant, with 21.3% of the variance of egoresilience being explained by these two variables.

**Table 3:** Model fitness index for the hypothesized model and the modified model

***Model fit measure***		***χ^2^(p)***	***DF***	***Δχ^2^***	***GFI***	***AGFI***	***CFI***	***NFI***	***CFI***	***RMR***	***RMSEA***
***Recommended value***		***(.05)***			***.90–1***	***.90–1***	***.90–1***	***.90–1***	***.90–1***	***.05 or less***	***.08 or less***
Hypothesized model	Total	269.838 (< .01)	21	12.849	.972	.940	.957	.953	.957	.053	.077
	Group 1	153.589 (<.01)	21	7.314	.953	.931	.953	.946	.953	.054	.080
	Group 2	142.568 (<.01)	21	6.789	.970	.937	.958	.952	.958	.053	.076
Modified model	Total	53.134 (< .01)	14	3.795	.993	.983	.991	.987	.991	.021	.038
	Group 1	35.342 (<.01)	14	2.524	.991	.977	.990	.983	.990	.024	.038
	Group 2	26.311 (.024)	14	1.879	.994	.983	.994	.988	.994	.018	.030

*Note:* Group 1: below average income, Group 2: above average income;

GFI: goodness-of-fit index, AGFI: adjusted goodness-of-fit index, NFI: normed fit index, CFI: comparative fit index, RMR: root mean-square residual, RMSEA: root mean squared error.

The predictor variables that directly and significantly affected adolescent life satisfaction were ego-resilience (β = .341) and positive parenting attitude (β = .318). In addition, the indirect effect through positive and negative parenting attitude on adolescents’ life satisfaction was .160 and .053, respectively. A mediating effect was found; however, the direct effect (β= −.022) and total effect (β= .031) were nonsignificant in the case of negative parenting attitude, while the total effect (β= .477) of positive parenting attitude was significant. These variables explained 31.4% of the variance of adolescents’ life satisfaction.

Positive parenting attitude was the only predictor variable that showed a significant influence (β = .095) on parents’ life satisfaction. The indirect effect on parents’ life satisfaction through positive and negative parenting attitude was nonsignificant, and the total effect (β = .113) of positive parenting attitude alone was significant. These variables explained 1.5% of the variance of parents’ life satisfaction (Table 3).

The path diagrams of both the below average income and average to above average income groups in a multi-group path analysis are shown in Figs. 2B and 2C. As was found in the full model, the predictor variables that directly and significantly affected ego-resilience were positive parenting attitude and negative parenting attitude for both income groups. Both predictor variables explained 20.1% and 22.9% of the variance of ego-resilience, respectively (Table 4).

**Table 4:** Multi-group path analysis according to income level

***Variables***		***Categories***	***SDE***	***SIE***	***STE***	***SMC***
Total	Ego-resilience	Positive parenting attitude	0.469^**^	-	0.469^**^	.213
		Negative parenting attitude	0.154^**^	-	0.154^**^	
	Life satisfaction of adolescent	Ego-resilience	0.341^**^	-	0.341^**^	.314
		Positive parenting attitude	0.318^**^	0.160^**^	0.477^**^	
		Negative parenting attitude	−0.022	0.053^**^	0.031	
	Life satisfaction of parent	Ego-resilience	0.038	-	0.038	.015
		Positive parenting attitude	0.095^**^	0.018	0.113^**^	
		Negative parenting attitude	−0.017	0.006	−0.011	
Below average income	Ego- resilience	Positive parenting attitude	0.441^**^	-	0.441^**^	.201
		Negative parenting attitude	0.144^**^	-	0.144^**^	
	Life satisfaction of adolescent	Ego-resilience	0.367^**^	-	0.367^**^	.284
		Positive parenting attitude	0.258^**^	0.162^**^	0.420^**^	
		Negative parenting attitude	−0.041	0.053^**^	0.012	
	Life satisfaction of parent	Ego-resilience	0.015	-	0.015	.027
		Positive parenting attitude	0.149^**^	0.006	0.155^**^	
		Negative parenting attitude	−0.034	0.002	−0.032	
Above average income	Ego- resilience	Positive parenting attitude	0.499^**^	-	0.499^**^	.229
		Negative parenting attitude	0.167^**^	-	0.167^**^	
	Life satisfaction of adolescent	Ego-resilience	0.303^**^	-	0.303^**^	.356
		Positive parenting attitude	0.398^**^	0.151^**^	0.550^**^	
		Negative parenting attitude	0.016	0.051^**^	0.066^**^	
	Life satisfaction of parent	Ego-resilience	0.066	-	0.066	.005
		Positive parenting attitude	−0.022	0.033	0.031	
		Negative parenting attitude	0.001	0.011	0.012	

*Note:* SDE: standardized direct effect, SIE: standardized indirect effect, STE: standardized total effect, SMC: squared multiple correlation;

* *P*<0.05,

** *P*<0.01.

As in the full model, the direct effect and the total effect of ego-resilience on adolescents’ life satisfaction were significant in both income groups. In the case of negative parenting attitude, only the indirect effect of ego-resilience was significant in the below average income group, while the indirect effect and total effect were significant in the equal to or above average income group. Negative parenting attitude mediated egoresilience on adolescents’ life satisfaction in average to above average income group. These variables explained 28.4% and 35.6% of the variance of adolescents’ life satisfaction, respectively (Table 4). Only the direct effect and total effect of positive parenting attitude on parents’ life satisfaction were significant in the below average income group, and ego-resilience, positive parenting attitude, negative parenting attitude were all nonsignificant in average to above average income group. These variables explained 2.7% and 0.5% of the variance of parents’ life satisfaction, respectively. Parents’ life satisfaction was significantly influenced only in the below average income group (Table 4).

## Discussion

Positive parenting attitude increased the egoresilience as well as the life satisfaction of both parents and adolescents, which is consistent with previous findings that positive parenting attitude increases ego-resilience (21) and parents’ life satisfaction (17). Parents’ negative parenting attitude influenced adolescents’ ego-resilience. Notably, it was positively correlated with over-expectation, which was inconsistent with the findings of a previous study (22) that reported no significant correlation between over-protection and egoresilience. This finding necessitates a closer examination of the relationship between egoresilience and negative parenting attitude by examining types of negative parenting in more detail in further studies.

Even though no significant difference in the influence of parenting attitude on adolescents’ life satisfaction by income level was found, parents’ life satisfaction was significantly influenced by positive parenting attitude when income was below average while parenting attitude was found to have no significant influence when income was average to above average.

Environmental factors such as socioeconomic status can also influence adolescents’ life satisfaction (28); however, school adjustment, self-esteem, interpersonal relationships (e.g., parents and peers) including school performance are more critical than anything else for adolescents’ satisfaction (29). Therefore, the influence of parenting attitude of parents on adolescents’ life satisfaction is not affected by income is consistent with previous studies that found that variables other than socioeconomic factors are playing a prominent role in adolescents’ life satisfaction.

Parents’ life satisfaction is affected by positive parenting attitude only when the income is below average. In other words, low-income parents increase their satisfaction as parents through positive parenting attitude, which leads to parents’ own life satisfaction. These results are consistent with those from a previous study (17) that stated the higher the parental role satisfaction, the higher the life satisfaction.

Even when considering income level, the influence of parents’ positive parenting attitude on adolescents’ life satisfaction is notable. In addition, parents’ life satisfaction is affected by positive parenting attitude for low-income parents. In the USA and the UK, negative parenting attitudes have been found to have a negative influence on depression, attention, and the quality of life of children (30,31). Asian parents have high expectations for academic performance and moral values but are ineffective and inadequate when communicating with their children (32).

Therefore, considering the situation in South Korea where the life satisfaction of early adolescents is the lowest among the OECD countries, and the fact that stress and depression among early adolescents are increasing (33), adopting positive parenting attitudes appears to be one of the appropriate ways to increase the life satisfaction of both parents and adolescents. Education and interventions are necessary for prospective parents or parents of a dysfunctional family. This will improve individuals’ ego-resilience and quality of life and become the basis for building stable families and society members.

## Conclusion

Positive parenting attitude was positively correlated with ego-resilience and the life satisfaction of parents and adolescents. Concerning negative parenting attitude, only over-expectation was positively correlated with ego-resilience, and in-consistency and intrusiveness were negatively correlated with adolescent life satisfaction. Although a nonsignificant difference between parenting attitude on adolescents’ life satisfaction by income level was found, parents’ life satisfaction was significantly influenced by positive parenting attitude when their income level was less than the average. Positive parenting attitude increases adolescents’ ego-resilience and the life satisfaction of parents and adolescents. We sought to use these results as the basic data for preparing strategies that improve the life satisfaction of parents and adolescents.

## Ethical considerations

Ethical issues (Including plagiarism, informed consent, misconduct, data fabrication and/or falsification, double publication and/or submission, redundancy, etc.) have been completely observed by the authors.
